# LncRNA LINC01026 Is Overexpressed in Psoriasis and Enhances Keratinocyte Cell Cycle Progression by Regulating the Ets Homologous Factor (EHF)

**DOI:** 10.1111/jcmm.70719

**Published:** 2025-07-16

**Authors:** Jingxia Lin, Hang Su, Yuanqiu Zhong, Hang Zheng, Yongfeng Chen

**Affiliations:** ^1^ Dermatology Hospital Southern Medical University Guangzhou Guangdong China

**Keywords:** cell cycle, ehf, keratinocyte, linc01206, long non‐coding RNA, psoriasis

## Abstract

Psoriasis is a chronic autoimmune skin disease characterised by a high recurrence rate and epidermal hyperproliferation. Recent studies have highlighted the pathogenic roles of long non‐coding RNAs (lncRNAs) in psoriasis. However, the cellular functions and underlying mechanisms of most lncRNAs remain largely unknown. In this study, we identified functional lncRNAs associated with cell cycle regulation through integrative analysis of RNA‐seq datasets from a psoriasis cohort. Interestingly, we observed significant upregulation of *LINC01206* in skin biopsies from psoriatic lesions, whereas its expression was downregulated following glucocorticoid treatment. Furthermore, we constructed a lncRNA‐protein‐coding gene (PCG) co‐expression network, which revealed that *LINC01206* tends to co‐express with cell cycle‐related genes, such as *CCNB1* and *CCNE1.* Using an in vitro keratinocyte model, we demonstrated that LINC01206 disrupts cell cycle progression, and its knockdown induced cell cycle arrest at the G0/G1 phase. Additional functional experiments showed that the expression of hyperproliferation‐associated keratins decreased upon *LINC01206* knockdown. Mechanistically, *LINC01206* promotes cell cycle progression at the G0/G1 phase by modulating the activity of Ets homologous factor (EHF). Our findings suggest that *LINC01206* enhances keratinocyte proliferation in psoriasis by regulating cell cycle progression, making it a potential therapeutic target for psoriasis treatment.

## 1 Introduction

Psoriasis is a chronic inflammatory skin disorder characterised by immune system dysregulation, leading to abnormal keratinocyte proliferation, excessive differentiation, and infiltration of inflammatory cells [1, 2, 3, 4, 5]. Keratinocytes, the primary cell type in the epidermis, play a central role in both the initiation and progression of psoriasis. Their growth and differentiation are tightly regulated by genetic, metabolic, and cell cycle mechanisms [6]. Additionally, transcription factors and non‐coding RNAs (ncRNAs) have been identified as key modulators of keratinocyte function and psoriasis pathogenesis [7, 8]. Given their critical role in disease development, keratinocytes are not only drivers of psoriasis but also represent a promising therapeutic target.

Long non‐coding RNAs (lncRNAs), a class of ncRNAs exceeding 200 nucleotides in length, have emerged as important regulators of biological processes such as cell proliferation, apoptosis, and inflammation [9, 10, 11]. Dysregulation of lncRNAs in keratinocytes has been implicated in psoriasis pathogenesis. For instance, lncRNA UCA1 promotes inflammatory responses and influences cell cycle progression in psoriatic keratinocytes [12], whereas lncRNA PRINS modulates psoriasis susceptibility by regulating apoptosis‐related proteins such as G1P3 [13]. These findings highlight the significance of lncRNAs in psoriasis and their potential as therapeutic targets. However, the functional mechanisms of many lncRNAs in psoriasis remain poorly understood, particularly their role in cell cycle regulation, which is a hallmark of keratinocyte hyperproliferation in the disease.

Although previous studies have highlighted the critical roles of mammalian lncRNAs in the diagnosis and treatment of human diseases, the functional mechanisms of many lncRNAs in psoriasis remain largely unexplored and poorly understood. Recent advancements, such as those reported in PMID:38111678 and PMID:39513037, have begun to shed light on the regulatory roles of lncRNAs in immune modulation and keratinocyte behaviour, yet significant gaps persist in our understanding of their precise molecular mechanisms in psoriasis pathogenesis [14, 15]. This underscores the need for further research to elucidate the functional contributions of lncRNAs, such as LINC01206, in psoriasis and their potential as therapeutic targets.

Therefore, this study aims to explore the potential roles of lncRNAs in psoriasis pathogenesis through an integrative analysis of RNA‐seq data from a psoriasis cohort, focusing on the interactions between lncRNAs and cell cycle‐related genes, as well as their regulatory mechanisms in keratinocyte proliferation and differentiation. Specifically, we seek to: (1) identify dysregulated lncRNAs in psoriatic lesions, (2) investigate their functional roles in keratinocyte cell cycle regulation, and (3) elucidate the molecular mechanisms underlying their involvement in psoriasis pathogenesis. By addressing these objectives, we aim to provide new insights into the role of lncRNAs in psoriasis and identify potential therapeutic targets for this chronic inflammatory skin disorder.

## 2 Materials and Methods

### 2.1 Data Collection

RNA‐seq datasets from both psoriasis patients and healthy controls were downloaded from the NCBI Gene Expression Omnibus (GEO) database under the accession number GSE54456, comprising a total of 174 samples, including 92 psoriatic and 82 healthy punch biopsies. Additionally, RNA‐seq datasets consisting of pre‐treatment, post‐treatment, and recurrent samples for each psoriasis patient were obtained from GSE114729. Raw sequencing reads were extracted from SRA files using the SRA‐toolkit.

### 2.2 RNA‐Seq Data Analysis

We employed a transcriptome analysis pipeline established in our previous studies. Briefly, low‐quality sequencing reads were filtered using adapter removal if the read length was less than 50 base pairs (bps) or the quality value was below 3. The remaining reads were then aligned to the human reference genome (hg19) using the STAR aligner [16]. Gene expression levels for all transcripts were calculated as fragments per kilobase of exon per million fragments mapped (FPKM) using Cufflinks. Differentially expressed genes (DEGs) were identified by comparing the psoriasis and healthy groups using DESeq2 [17]. Genes were considered differentially expressed if they met the following criteria: a Benjamini–Hochberg adjusted *p* < 0.05 and | Log2 (fold‐change) | ≥ 0.58.

### 2.3 Functional Enrichment Analysis

The differentially expressed genes were analysed using the DAVID functional annotation pipeline for gene function enrichment analysis. KEGG pathways were considered significant if the adjusted *p*‐value was less than 0.05. Additionally, a pre‐ranked gene list on the basis of log2 (fold‐change) was analysed using the GSEA package against the MSigDB database [18, 19].

### 2.4 Construction of the lncRNA‐PCG Co‐Expression Network

A gene expression matrix was constructed by integrating gene expression profiles from 174 RNA‐seq datasets, including 92 psoriatic and 82 healthy punch biopsies. We then applied Weighted Gene Co‐expression Network Analysis (WGCNA) to construct a gene co‐expression network using the established gene expression matrix as input [20, 21]. Weak co‐expression gene pairs were filtered out using an adjacency threshold greater than 0.02 and requiring interaction with at least one lncRNA molecule, resulting in a reliable lncRNA‐PCG (protein‐coding gene) co‐expression network [22].

### 2.5 Construction of the TF‐mRNA Network

WGCNA was employed to construct a gene co‐expression network using the established gene expression matrix as input [21]. Weak co‐expression gene pairs were filtered out using an adjacency threshold greater than 0.02. The resulting reliable LINC01206‐gene co‐expression network was imported into Cytoscape for visualisation.

### 2.6 Collection of Clinical Specimens

This study was approved by the Ethics Committee of the Dermatology Hospital of Southern Medical University. Lesion samples were collected from 12 psoriasis vulgaris patients who met the following inclusion criteria: (1) clinical and histopathological confirmation of psoriasis vulgaris, (2) no systemic or topical treatment for at least 4 weeks, and (3) absence of other inflammatory skin conditions. Additionally, 10 healthy control skin tissue samples were obtained from age‐ and sex‐matched individuals undergoing elective plastic surgery, with no history of inflammatory or autoimmune diseases. All specimens were snap‐frozen in liquid nitrogen and stored at –80°C for RNA extraction.

### 2.7 Cell Culture

Primary normal human keratinocytes (NHEK; BNCC, Cat. No. 3405CA93) were cultured in Dulbecco's Modified Eagle's Medium (DMEM) supplemented with 10% fetal bovine serum (FBS). NHEK cells were treated with 100 ng/μL of IL‐17A. At specified time points following cytokine exposure, cells were harvested for quantification of LINC01206 expression. All cells were maintained at 37°C in a humidified atmosphere with 5% CO_2_.

### 2.8 shRNAs and Transfection

shRNAs were used according to the manufacturer's instructions provided with the transfection kit. Transfected NHEK cells were cultured in DMEM containing 1 μg/mL puromycin and 10% FBS. The shRNA interference sequences targeting LINC01206 are listed in Table S4.

### 2.9 Quantitative Real‐Time PCR

Total RNA from NHEK cells or skin tissues was extracted using TRIzol RNA Isolation reagent (ThermoFisher Cat. NO. 191005) according to the manufacturer's instructions. The expression of genes was quantified by Real‐time PCR Mixture Assays with SYBR Green Premix Pro Taq HS qPCR Kit (Cat. NO. AG11701) using GAPDH as a control. The qPCR reactions were performed using the following thermal cycling protocol: initial denaturation at 95°C for 30 s, followed by 40 cycles of denaturation at 95°C for 5 s and annealing/extension at 60°C for 30 s. Melt curve analysis was performed at the end of each run to verify the specificity of amplification. All primers used in this study are listed in Table S5.

### 2.10 Western Blotting

CCNB1 and CCNE1 antibodies were purchased from Abcam. The primary antibody concentrations used were 1:2000 for CCNB1 (Item No. ab32053) and 1:1000 for CCNE1 (Item No. ab33911).

### 2.11 Flow Cytometry Analysis Cell Cycle

NHEK cells transfected with shRNAs were cultured to 90% confluence in six‐well plates, washed with cold phosphate‐buffered saline (PBS), fixed with absolute ethanol, and stored at −20°C until analysis. Prior to flow cytometry, cells were rehydrated with PBS, stained with DNA staining solution for 30 min at room temperature, and analysed using a BD FACS Celesta flow cytometer.

### 2.12 Statistical Analysis

Data analysis and visualisation were performed using GraphPad Prism 8.0 (GraphPad Software, San Diego, CA, USA). Differences between two independent groups were assessed using Student's *t*‐test or the Mann–Whitney *U* test, as appropriate. Results are presented as the mean ± standard deviation (Mean ± SD) of three independent experiments. A *p*‐value < 0.05 was considered statistically significant.

## 3 Results

### 3.1 Cell Cycle Dysregulation in Psoriasis

**FIGURE 1:**
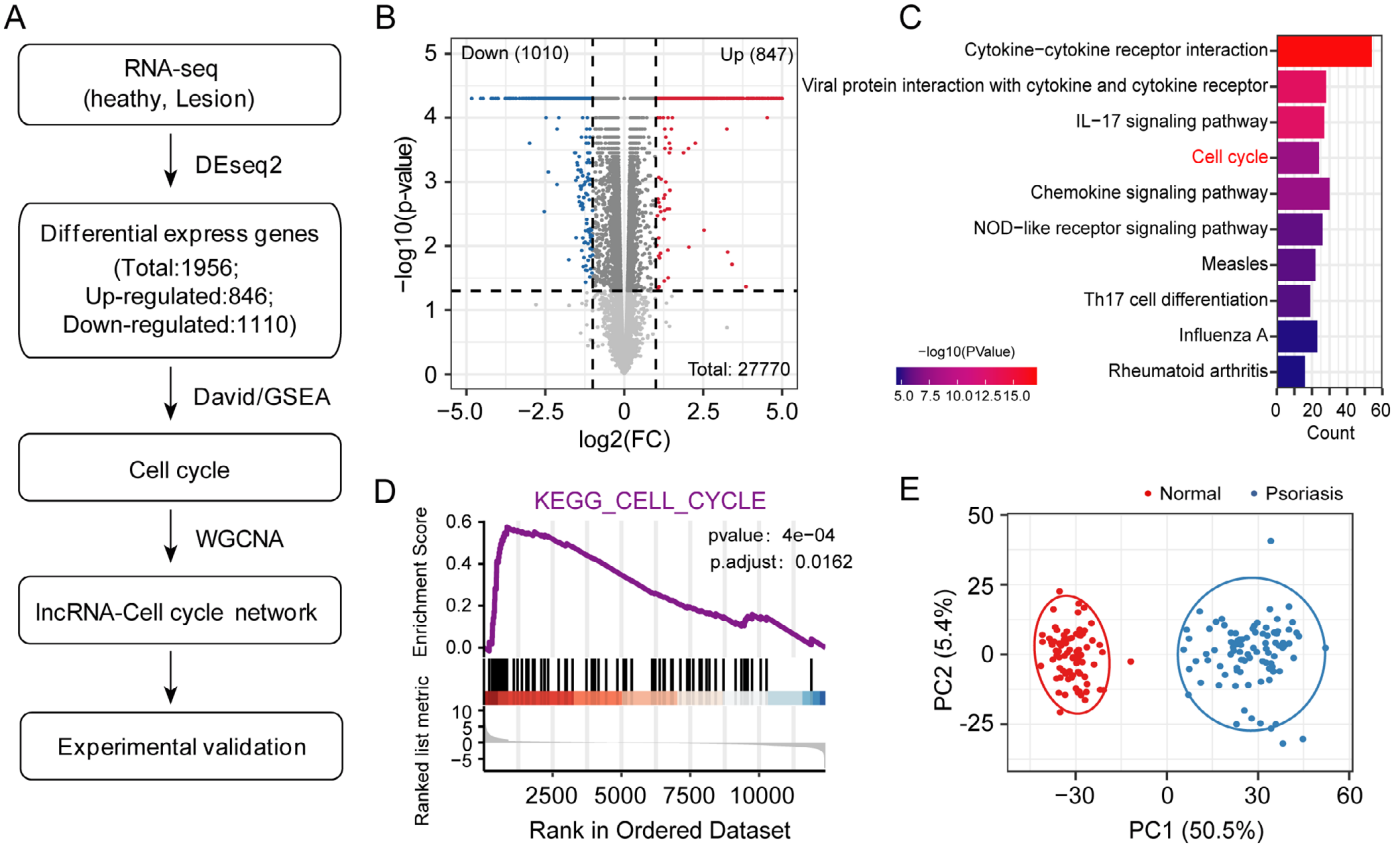
Dysregulation of the cell cycle associated with psoriasis. (A) Overview of RNA‐seq processing pipeline used in this study. (B) Volcano plot showing the differentially expressed genes (DEGs) in psoriasis compared with the healthy group. Upregulated genes are highlighted in red, whereas downregulated genes are highlighted in blue. (C) KEGG pathway enrichment analysis for upregulated differentially expressed genes in psoriasis compared with the healthy group. (D) Gene Set Enrichment Analysis showed that upregulated genes are associated with the JAK/STAT signalling pathway in psoriasis. (E) Principal component analysis (PCA) showed that cell cycle‐associated genes distinguished psoriatic skin (in blue) from healthy skin (in red).

**FIGURE 2:**
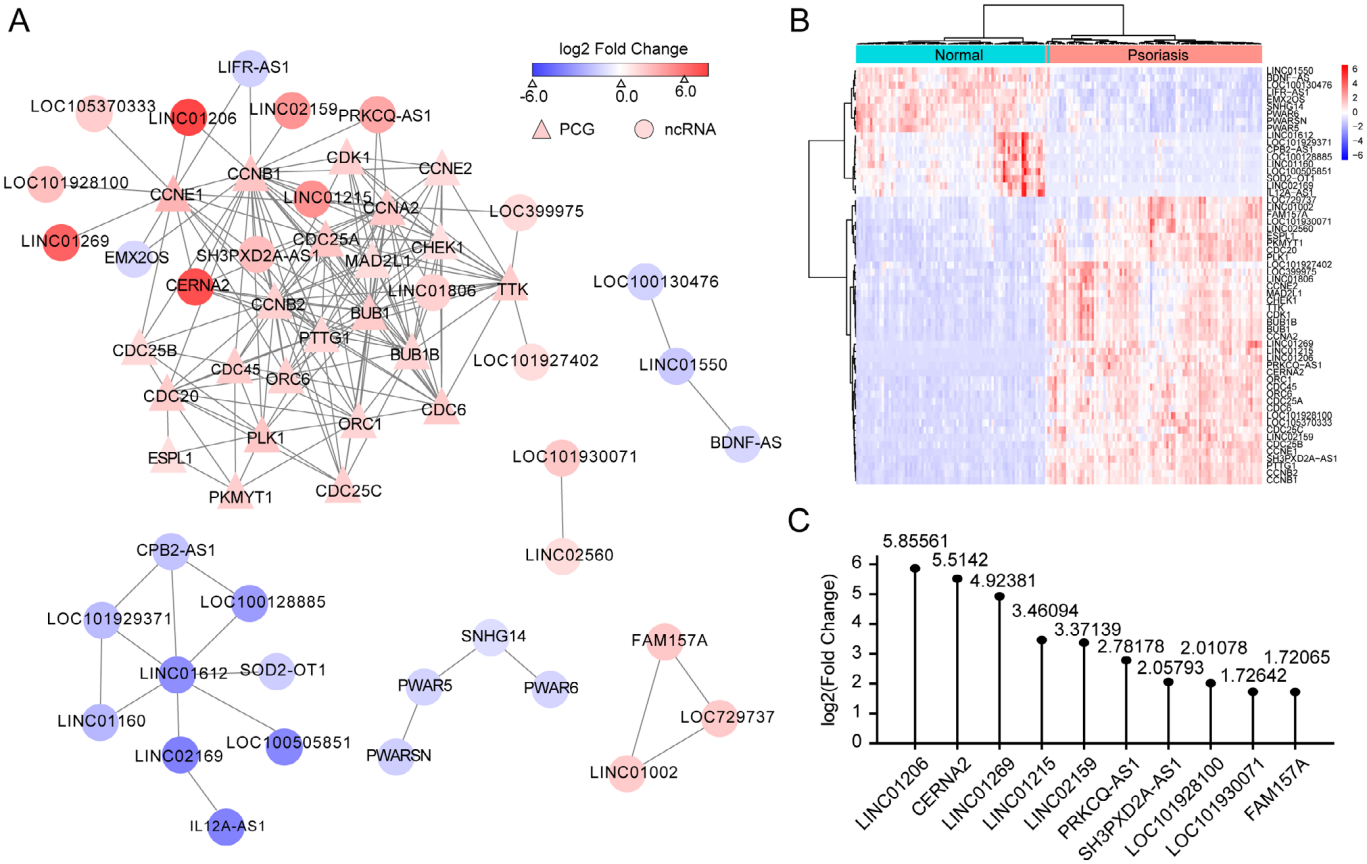
LncRNA LINC01206 participates in the cell cycle pathway of psoriasis. (A) The mRNA‐lncRNA co‐expression network involved in the cell cycle pathway in psoriasis. The network consists of 62 edges among 29 lncRNA (square) and 29 mRNAs (circle). The gradient of red and blue represents the log2 (fold‐change) of upregulated and downregulated genes, respectively. (B) Heatmap showing the DEGs associated with the cell cycle in psoriasis. (C) Top10 lncRNAs significantly differentially expressed in psoriasis that co‐expressed with key factors in cell cycle progression include LINC01260, CERNA2, LINC01269, LINC01215, LINC02159, PRKCQ‐AS1, SH3PXD2A‐AS1, LOC101928100, LOC101930071, and FAM157A.

Previous studies have demonstrated that psoriasis is characterised by aberrant keratinocyte differentiation and proliferation, with dysregulation of cell cycle arrest and epidermal differentiation‐associated genes [23]. However, the role of lncRNAs in keratinocyte proliferation remains poorly understood [12]. To address this, we re‐analysed RNA‐seq data from 82 normal skin samples and 92 psoriatic lesions. We identified 846 upregulated and 1110 downregulated genes in psoriatic skin compared to normal skin (Figure 1B and Table S1). KEGG enrichment analysis revealed that upregulated genes were significantly associated with cell cycle progression (Figure 1C). Gene set enrichment analysis (GSEA) further confirmed elevated expression of cell cycle‐associated genes in psoriatic skin (*p* < 0.001) (Figure 1D and Table S2), suggesting a critical role for cell cycle dysregulation in psoriasis. Principal component analysis (PCA) using cell cycle‐related genes clearly distinguished psoriatic skin from normal skin (Figure 1E), and hierarchical clustering analysis of cell cycle gene expression profiles supported these findings. Notably, key cell cycle regulators such as *CCNB1*, *CCNE1*, and *CDK1* were significantly upregulated in psoriatic skin (Figure 2B). These results collectively demonstrate that cell cycle dysregulation plays a pivotal role in the cellular progression of psoriasis.

### 3.2 LncRNA LINC01206 Regulates the Cell Cycle Pathway in Psoriasis

LncRNAs are known to participate in various biological processes in disease [24]. We hypothesised that differentially expressed lncRNAs may regulate the cell cycle in psoriasis. To explore this, we constructed an lncRNA‐protein‐coding gene (PCG) co‐expression network using weighted gene co‐expression network analysis (WGCNA). This analysis revealed strong interactions between lncRNAs and cell cycle genes. Specifically, *LINC02159* showed a positive correlation with *CCNB1*, whereas *LINC01206* was positively correlated with both *CCNB1* and *CCNE1* (Figure 2A and Table S3), suggesting that upregulated lncRNAs may coordinate with cell cycle genes to drive psoriasis progression. Heatmap analysis of differentially expressed lncRNAs and cell cycle genes further distinguished psoriatic skin from normal skin (Figure 2B), highlighting the functional relevance of these lncRNAs. Additionally, the expression of 10 selected lncRNAs, including *LINC01206*, was significantly upregulated in psoriatic skin (Figure 2C), implicating their potential role in cell cycle regulation. These findings suggest that *LINC01206* may be a key regulator of the cell cycle pathway in psoriasis.

### 3.3 LINC01206 as a Potential Therapeutic Target in Psoriasis

**FIGURE 3:**
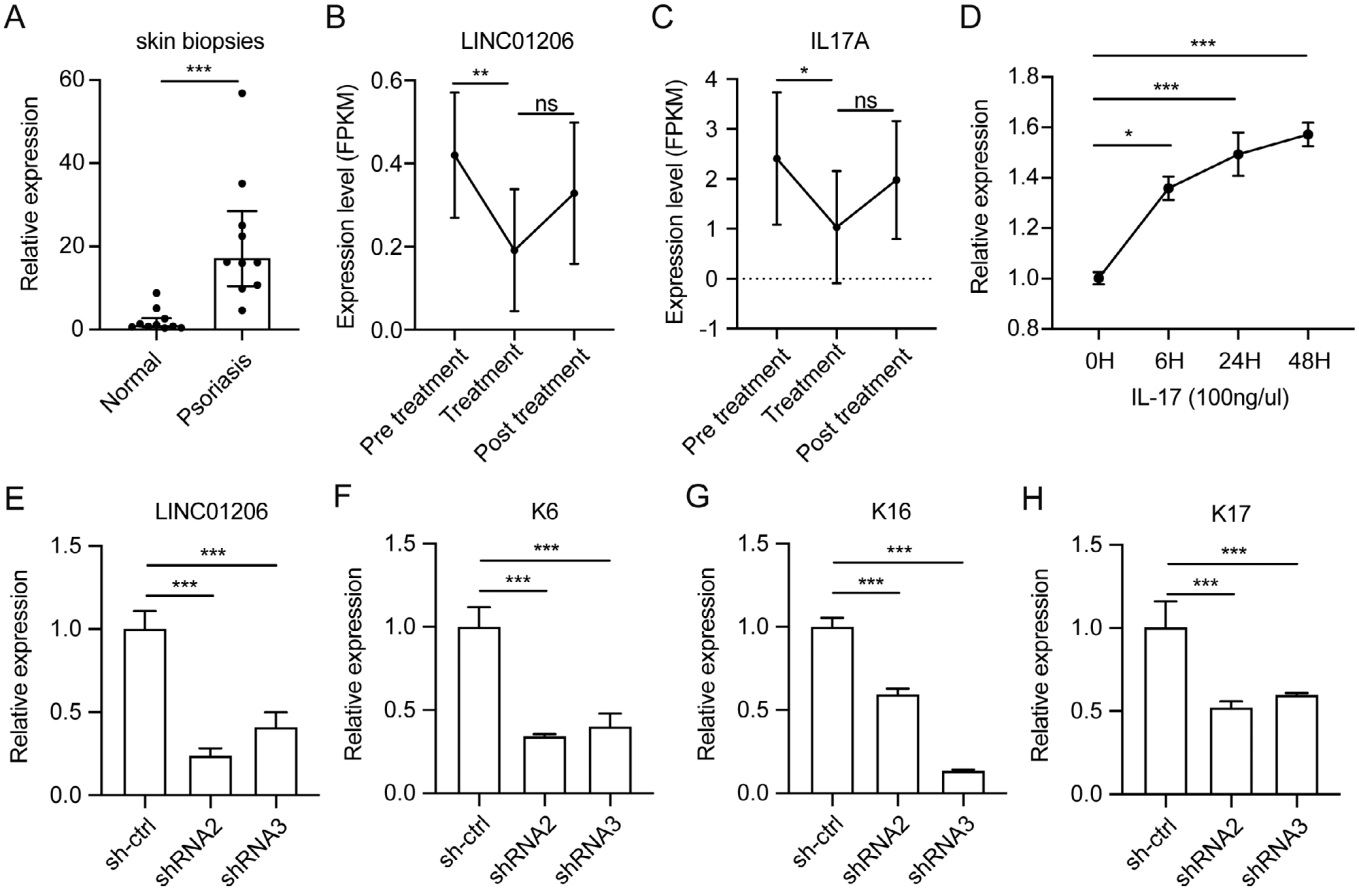
LINC01206 induces hyperproliferation in keratinocytes and serves as a potential therapy target site in psoriasis. (A) The expression of LINC01206 was determined by real‐time PCR (RT‐PCR) on clinical samples. LINC206 was significantly elevated in skin biopsy samples (*p* = 0.0010). (B‐C) Re‐analysis of a set of RNA‐seq data (GSE114729). The expression of LINC01206 (*p* = 0.0029) (B) and IL‐17A (*p* = 0.0223) (C) in psoriasis before and after treatment with a glucocorticoid. (D) The expression levels of LINC01206 in NHEK cells treated with IL‐17A (100 ng/mL) stimulated at the indicated time points (6, 24 or 48 h). (E‐H) RT‐qPCR analysis of the expression of LINC01206 (E), KRT6 (F), KRT16 (G), and KRT17 (H) in NHEK cells transfected with shRNA2 or shRNA3. All experiments were performed in triplicate, and the average expression level was reported. Significant differences were determined using the Mann–Whitney unpaired two‐tailed *t*‐tests. (* indicates significance, *p* < 0.05; ** indicates significance, *p* < 0.01; *** indicates significance, *p* < 0.001).

To validate our findings, we performed qPCR on skin biopsies from psoriasis patients and healthy volunteers. *LINC01206* expression was markedly elevated in psoriatic lesions compared to normal skin (*p* = 0.0010) (Figure 3A). Intriguingly, RNA‐seq analysis revealed that *LINC01206* expression was significantly reduced following glucocorticoid treatment (*p* = 0.0029), suggesting its potential as a therapeutic target (Figure 3B). Consistent with this, *IL‐17A* expression was also dramatically decreased after glucocorticoid treatment (*p* = 0.0223) (Figure 3C). These results demonstrate that *LINC01206* is upregulated in psoriatic lesions and downregulated by glucocorticoid therapy, supporting its role as a potential therapeutic target in psoriasis.

### 3.4 Knockdown of *LINC01206* Inhibits Keratinocyte Proliferation

To investigate the functional impact of *LINC01206* in keratinocytes, we established a psoriatic keratinocyte model by stimulating NHEK cells with IL‐17A. As expected, *LINC01206* expression was significantly upregulated in NHEK cells following IL‐17A stimulation (Figure 3D), consistent with its expression pattern in clinical psoriasis samples. We then generated stable *LINC01206* knockdown NHEK cell lines using two independent shRNAs (Figure 3E). QPCR analysis revealed that knockdown of *LINC01206* led to increased expression of hyperproliferation markers *KRT6*, *KRT16*, and *KRT17* (Figure 3F–H), suggesting that *LINC01206* may promote aberrant keratinocyte proliferation in psoriasis.

### 3.5 *LINC01206* Knockdown Induces G0/G1 Phase Cell Cycle Arrest

**FIGURE 4:**
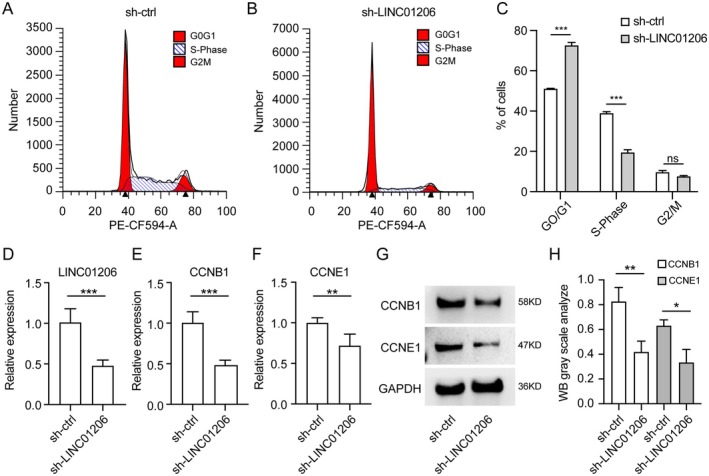
Knockdown of LINC01206 induces cell cycle arrest in G0/G1 phase. (A–C) Cell cycle phase distribution analysis through flow cytometry. (A, B) Flow cytometry analysis of control and LINC01206‐specific knockdown group cell cycles. Left‐sided red peaks represent the percentage of cells in the G0/G1 phase, the right‐sided red peaks represent the percentage of cells in the G2/M phase, and the shaded bottoms represent the percentage of cells in the S phase. (C) Quantified flow cytometry results, indicating the proportion of cells in the G0/G1, S, and G2/M phases of the cell cycle. **p < 0.01 vs. the control. Data are presented as the mean ± standard deviation and are representative of three independent experiments with similar results. (D‐F) RT‐qPCR analysis of the expression of LINC01206 (*p* < 0.0010) (D), CCNB1 (E), and CCNE1 (F) in NHEK cells transfected with shRNA. (G) Western blot analysis of CCNB1 and CCNE1 protein levels in control or LINC01206‐specific knockdown cells. (H) Statistical analysis for the Western blot data. All experiments were performed in triplicate, and the average expression level was reported. Significant differences were determined using the Mann–Whitney unpaired two‐tailed t‐tests. (* indicates significance, *p* < 0.05; ** indicates significance, *p* < 0.01; *** indicates significance, *p* < 0.001).

Cell cycle regulation is critical for controlling cell proliferation [25]. Flow cytometry analysis of *LINC01206* shRNA knockdown cells revealed a significant decrease in S‐phase cells and an increase in G0/G1‐phase cells (Figure 4D), indicating that *LINC01206* knockdown inhibits cell cycle progression by inducing G0/G1‐phase arrest (Figure 4A–C). Cyclin E, a key regulator of the G1‐S transition, and cyclin B, which controls the G2‐M transition, were found to be co‐expressed with *LINC01206*. Knockdown of *LINC01206* led to decreased expression of *CCNB1* and *CCNE1* at both mRNA and protein levels (Figure 4D–G). Quantitative analysis of Western blot data demonstrated that the protein levels of CCNB1 and CCNE1 were reduced by 58% and 66%, respectively, in *LINC01206* knockdown cells compared to control cells (Figure 4H). These results further support the role of LINC01206 in regulating cell cycle progression and keratinocyte proliferation.

### 3.6 *LINC01206* Enhances Cell Cycle Progression via *EHF* Regulation

**FIGURE 5:**
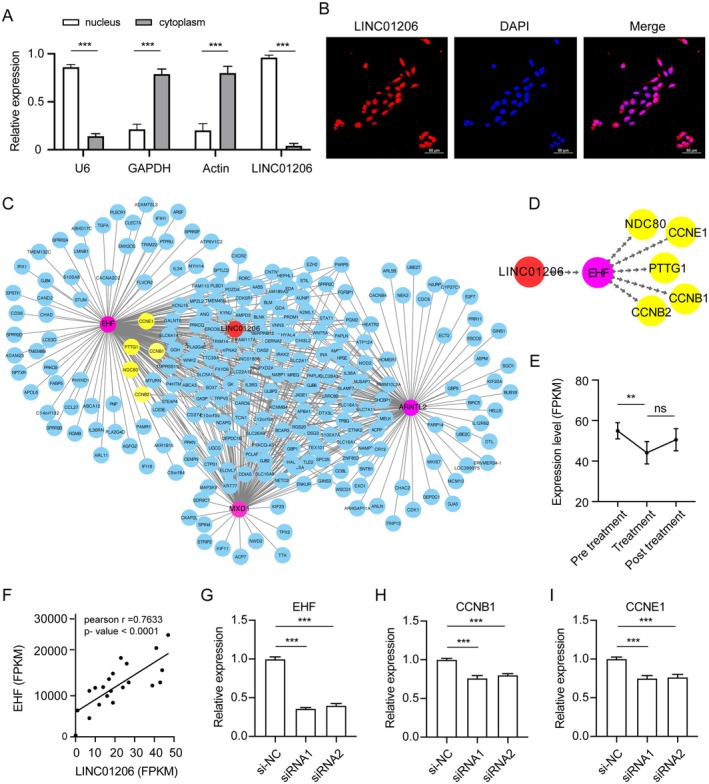
LINC01206 enhances the cell cycle progression via regulating EHF. (A) RT‐qPCR detects the expression of LINC01206 in nuclear RNA and cytoplasmic RNA. Nuclear control: U6. Cytoplasmic control: GAPDH, β‐actin. Values were calculated relative to total expression and are shown as the average and standard error of three independent experiments. (B) Fluorescence in situ hybridisation detects the location of LI01206 in NHEK cells. Nuclear control: U6. Cytoplasmic control: 18 s. (C) Overview of TF‐LINC01206‐mRNA interaction network. Three TFs associated with LINC01206 interacted with 433 TF‐target genes. (D) The EHF‐LINC01206 co‐expression network. (E) The expression of EHF in psoriasis before and after treatment according to RNA‐seq data (GSE114729). (F) (G‐I) RT‐qPCR detects the expression of EHF (G), CCNB1 (H) and CCNE1 (I) in EHF‐siRNA cells. *** *p* < 0.001.

To elucidate the functional role of *LINC01206*, we separated cytoplasmic and nuclear RNA from keratinocytes. QPCR assays confirmed that *LINC01206* is predominantly localised in the nucleus (Figure 5A), a finding corroborated by fluorescence in situ hybridisation (FISH) (Figure 5B). Given that nuclear lncRNAs often regulate transcription, we constructed a *LINC01206*‐TF‐gene network using WGCNA analysis and identified three transcription factors (TFs)‐EHF, ARNTL2, and MXD1 as potential regulators (Figure 5C). Notably, EHF‐target genes, including *CCNB1*, *CCNB2*, and *CCNE1*, are involved in cell cycle checkpoints (Figure 5D). RNA‐seq data (GSE114729) showed that *EHF* is upregulated in psoriatic lesions and downregulated after treatment (Figure 5E). Co‐expression analysis revealed a strong correlation between *LINC01206* and *EHF* in psoriasis (Figure 5F), suggesting their functional interaction. SiRNA knockdown of *EHF* in NHEK cells significantly reduced the expression of *CCNB1* and *CCNE1* (Figure 5G–I), indicating that *LINC01206* enhances cell cycle progression by targeting *CCNB1* and *CCNE1* through *EHF* regulation.

These findings provide robust functional evidence that *LINC01206* plays a critical role in keratinocyte proliferation and cell cycle progression in psoriasis. By regulating *EHF* and its downstream targets, *LINC01206* emerges as a key player in psoriasis pathogenesis and a potential therapeutic target for future interventions.

## 4 Discussion

Psoriasis is a chronic inflammatory skin disorder marked by epidermal hyperplasia and immune cell infiltration, with a high prevalence and significant comorbidities [26, 27]. The pathogenesis of psoriasis is multifactorial, involving genetics, cytokines, metabolism, cell signalling, transcription factors, and non‐coding RNAs (ncRNAs) [10, 28]. Among ncRNAs, long non‐coding RNAs (lncRNAs) have emerged as critical regulators in psoriasis, as evidenced by transcriptomic analyses of psoriatic skin [9, 29]. This study investigates the role of lncRNAs, particularly LINC01206, in psoriasis pathogenesis, focusing on their involvement in cell cycle regulation. Through bioinformatics analysis and experimental validation, we demonstrate that LINC01206 plays a pivotal role in psoriasis by modulating cell cycle progression, offering new insights into the disease's molecular mechanisms and potential therapeutic targets.

Our findings reveal that upregulated genes in psoriasis are significantly enriched in the cell cycle pathway. Through lncRNA‐protein‐coding gene (PCG) co‐expression analysis, we identified interactions between *LINC01206* and key cell cycle regulatory genes, such as *CCNB1* and *CCNE1*. Experimental validation confirmed that LINC01206 is elevated in psoriatic lesions and promotes keratinocyte proliferation and cell cycle progression [30, 31]. Further mechanistic investigations using transcription factor (TF) target screening revealed that *LINC01206* and cell cycle‐associated genes are co‐regulated by the transcription factor EHF. This regulatory circuit was experimentally validated in normal human epidermal keratinocytes (NHEK), highlighting the critical role of *LINC01206* in psoriasis pathogenesis through its interaction with EHF and cell cycle genes.

The mammalian cell cycle is a tightly regulated process essential for controlling cell proliferation, and its dysregulation is a hallmark of psoriasis, characterised by keratinocyte hyperproliferation [25, 32, 33]. Our study supports a network model in which lncRNAs and proteins collaboratively disrupt cell cycle progression in psoriasis [34]. Specifically, *LINC01206* interacts with *CCNB1* and *CCNE1*, genes central to cell cycle regulation. Additionally, we identified EHF, a member of the Ets family of transcription factors, as a key regulator of epidermal homeostasis and cell cycle progression. EHF promotes the expression of cell cycle‐associated genes such as *CCNB1*, *CCND1*, and *PCNA*, whereas also activating genes like *p21*. These findings establish a novel lncRNA‐TF‐mRNA regulatory network, providing a mechanistic framework for understanding how *LINC01206* enhances cell cycle progression in psoriasis [22, 35, 36].

To further elucidate the role of LINC01206 in psoriasis, we examined its expression in psoriatic lesions and IL‐17A‐stimulated NHEK cells. IL‐17A is a primary driver of skin pathology in psoriasis, and our results showed that LINC01206 expression is significantly upregulated in both psoriatic lesions and IL‐17A‐stimulated keratinocytes. This suggests that *LINC01206* is activated in inflammatory skin environments and contributes to the dysregulation of cell cycle progression [11]. Moreover, we observed that knockdown of *LINC01206* in NHEK cells led to decreased expression of keratinocyte hyperproliferation markers, such as K6, K16, and K17 [37]. These findings indicate that *LINC01206* modulates keratinocyte proliferation by regulating key cell cycle genes, ultimately contributing to the hyperproliferative phenotype characteristic of psoriasis.

Although this study provides valuable insights into the role of LINC01206 in psoriasis pathogenesis by integrating bioinformatics analyses, such as RNA‐seq re‐analysis, KEGG enrichment, and gene set enrichment analysis (GSEA), and establishing a novel lncRNA‐PCG co‐expression network using weighted gene co‐expression network analysis (WGCNA) to reveal strong interactions between LINC01206 and cell cycle genes like *CCNB1* and *CCNE1*, several limitations must be acknowledged. The study primarily relies on in vitro models and skin biopsy samples, which may not fully capture the complexity of the in vivo environment, and the absence of in vivo animal models limits our understanding of the broader physiological context of LINC01206. Additionally, although we identified strong interactions between *LINC01206* and cell cycle genes, the precise molecular mechanisms, such as direct binding between *LINC01206* and the transcription factor *EHF*, require further investigation. Furthermore, the sample size for assessing glucocorticoid treatment effects is relatively small, and the study lacks stratification on the basis of disease severity or treatment duration, which could affect the generalisability of the findings. Finally, although our focus was on cell cycle regulation, the multifactorial nature of psoriasis suggests that LINC01206 may influence other cellular processes and signalling pathways, warranting further exploration. Despite these limitations, the study makes significant contributions by validating the upregulation of *LINC01206* in psoriatic lesions and its downregulation following glucocorticoid treatment, suggesting its potential as a therapeutic target, and by mechanistically linking *LINC01206* to *EHF*, revealing a previously unknown pathway in psoriasis pathogenesis.

In conclusion, this study enhances our understanding of the role of LINC01206 in psoriasis by demonstrating its involvement in cell cycle regulation and keratinocyte hyperproliferation. These findings not only advance our knowledge of psoriasis pathogenesis but also open new avenues for therapeutic interventions targeting lncRNAs. Future studies should focus on validating these findings in vivo and exploring the broader regulatory roles of LINC01206 in psoriasis and other inflammatory skin diseases.

## Author Contributions

**Jingxia Lin:** funding acquisition (equal), investigation (equal), methodology (equal), writing – original draft (lead). **Hang Su:** validation (equal), visualization (equal), writing – original draft (equal). **Yuanqiu Zhong:** formal analysis (equal), investigation (equal), methodology (equal), validation (equal). **Hang Zheng:** formal analysis (equal), validation (equal). **Yongfeng Chen:** conceptualization (lead), funding acquisition (lead), methodology (equal), writing – review and editing (lead).

## Ethics Statement

Ethical approval for this study was obtained from the Dermatology Hospital of Southern Medical University Office of Medical Ethics Institutional Review Board (APPROVAL NUMBER 2021077).

## Conflicts of Interest

The authors declare no conflicts of interest.

## Supporting information


**Table S1** List of differentially expressed genes (DEGs) used in this study.
**Table S2:** The enriched KEGG pathway of upregulated DEGs.
**Table S3:** LncRNA and mRNA co‐expression network.
**Table S4:** List of shRNA interference sequences of LINC01206 used in this study.
**Table S5:** List of primers used in this study.

## Data Availability

The data that supports the findings of this study are available in the supporting information of this article.

## References

[1] S. K. Ippagunta , R. Gangwar , D. Finkelstein , et al., “Keratinocytes Contribute Intrinsically to Psoriasis Upon Loss of Tnip1 Function,” Proceedings of the National Academy of Sciences of the United States of America 113 (2016): E6162–E6171. 27671649 10.1073/pnas.1606996113 PMC5068263

[2] I. S. Afonina , E. Van Nuffel , and R. Beyaert , “Immune Responses, and Therapeutic Options in Psoriasis,” Cellular and Molecular Life Sciences 78 (2021): 2709–2727. 33386888 10.1007/s00018-020-03726-1 PMC11072277

[3] C. Albanesi , S. Madonna , P. Gisondi , and G. Girolomoni , “The Interplay Between Keratinocytes and Immune Cells in the Pathogenesis of Psoriasis,” Frontiers in Immunology 9 (2018): 1549. 30034395 10.3389/fimmu.2018.01549 PMC6043636

[4] A. W. Armstrong and C. Read , “Pathophysiology, Clinical Presentation, and Treatment of Psoriasis: A Review,” JAMA 323 (2020): 1945–1960. 32427307 10.1001/jama.2020.4006

[5] D. Yan , A. Blauvelt , A. K. Dey , et al., “New Frontiers in Psoriatic Disease Research, Part II: Comorbidities and Targeted Therapies,” Journal of Investigative Dermatology 141 (2021): 2328–2337. 33888321 10.1016/j.jid.2021.02.743 PMC8464483

[6] M. Xu , H. Lu , Y.‐H. Lee , et al., “An Interleukin‐25‐Mediated Autoregulatory Circuit in Keratinocytes Plays a Pivotal Role in Psoriatic Skin Inflammation,” Immunity 48 (2018): 787–798.e4. 29653697 10.1016/j.immuni.2018.03.019

[7] A. Zolotarenko , E. Chekalin , A. Mesentsev , et al., “Integrated Computational Approach to the Analysis of RNA‐Seq Data Reveals New Transcriptional Regulators of Psoriasis,” Experimental & Molecular Medicine 48 (2016): e268. 27811935 10.1038/emm.2016.97 PMC5133374

[8] C. Antonatos , K. Grafanaki , P. Asmenoudi , et al., “Contribution of the Environment, Epigenetic Mechanisms and Non‐Coding RNAs in Psoriasis,” Biomedicine 10 (2022): 1934. 10.3390/biomedicines10081934 PMC9405550 36009480

[9] L. C. Tsoi , M. K. Iyer , P. E. Stuart , et al., “Analysis of Long Non‐Coding RNAs Highlights Tissue‐Specific Expression Patterns and Epigenetic Profiles in Normal and Psoriatic Skin,” Genome Biology 16 (2015): 24. 25723451 10.1186/s13059-014-0570-4 PMC4311508

[10] P. J. Batista and H. Y. Chang , “Long Noncoding RNAs: Cellular Address Codes in Development and Disease,” Cell 152 (2013): 1298–1307. 23498938 10.1016/j.cell.2013.02.012 PMC3651923

[11] P. Malakar , S. Shukla , M. Mondal , R. K. Kar , and J. A. Siddiqui , “The Nexus of Long Noncoding RNAs, Splicing Factors, Alternative Splicing and Their Modulations,” RNA Biology 21 (2024): 1–20. 10.1080/15476286.2023.2286099 PMC10761143 38017665

[12] Y. Hu , L. Lei , L. Jiang , et al., “LncRNA UCA1 Promotes Keratinocyte‐Driven Inflammation via Suppressing METTL14 and Activating the HIF‐1α/NF‐κB Axis in Psoriasis,” Cell Death & Disease 14 (2023): 279. 37076497 10.1038/s41419-023-05790-4 PMC10115875

[13] K. Szegedi , E. Sonkoly , N. Nagy , et al., “The Anti‐Apoptotic Protein G1P3 Is Overexpressed in Psoriasis and Regulated by the Non‐Coding RNA,” PRINS. Exp Dermatol 19 (2010): 269–278. 20377629 10.1111/j.1600-0625.2010.01066.x

[14] Y. Gao , J. Li , M. Ma , et al., “Prognostic Prediction of m6A and Ferroptosis‐Associated lncRNAs in Liver Hepatocellular Carcinoma,” J Transl Int Med 12 (2024): 526–529. 39513037 10.1515/jtim-2024-0023 PMC11538885

[15] A. Ghasemian , H. A. Omear , Y. Mansoori , et al., “Long Non‐Coding RNAs and JAK/STAT Signaling Pathway Regulation in Colorectal Cancer Development,” Frontiers in Genetics 14 (2023): 1297093. 38094755 10.3389/fgene.2023.1297093 PMC10716712

[16] A. Dobin , C. A. Davis , F. Schlesinger , et al., “STAR: Ultrafast Universal RNA‐Seq Aligner,” Bioinformatics 29 (2013): 15–21. 23104886 10.1093/bioinformatics/bts635 PMC3530905

[17] M. I. Love , W. Huber , and S. Anders , “Moderated Estimation of Fold Change and Dispersion for RNA‐Seq Data With DESeq2,” Genome Biology 15 (2014): 550. 25516281 10.1186/s13059-014-0550-8 PMC4302049

[18] M. Kanehisa , M. Furumichi , M. Tanabe , Y. Sato , and K. Morishima , “KEGG: New Perspectives on Genomes, Pathways, Diseases and Drugs,” Nucleic Acids Research 45 (2017): D353–D361. 27899662 10.1093/nar/gkw1092 PMC5210567

[19] A. Subramanian , H. Kuehn , J. Gould , P. Tamayo , and J. P. Mesirov , “GSEA‐P: A Desktop Application for Gene Set Enrichment Analysis,” Bioinformatics 23 (2007): 3251–3253. 17644558 10.1093/bioinformatics/btm369

[20] P. Langfelder and S. Horvath , “WGCNA: An R Package for Weighted Correlation Network Analysis,” BMC Bioinformatics 9 (2008): 559. 19114008 10.1186/1471-2105-9-559 PMC2631488

[21] G. Pei , L. Chen , and W. Zhang , “WGCNA Application to Proteomic and Metabolomic Data Analysis,” Meth Enzymol 585 (2017): 135–158. 10.1016/bs.mie.2016.09.016 28109426

[22] H. Li , C. Yang , J. Zhang , W. Zhong , L. Zhu , and Y. Chen , “Identification of Potential Key mRNAs and LncRNAs for Psoriasis by Bioinformatic Analysis Using Weighted Gene Co‐Expression Network Analysis,” Molecular Genetics and Genomics 295 (2020): 741–749. 32125527 10.1007/s00438-020-01654-0

[23] E. Palazzo , M. D. Kellett , C. Cataisson , et al., “A Novel DLX3‐PKC Integrated Signaling Network Drives Keratinocyte Differentiation,” Cell Death and Differentiation 24 (2017): 717–730. 28186503 10.1038/cdd.2017.5 PMC5384032

[24] X. Lin , Y. Lu , C. Zhang , et al., “LncRNADisease v3.0: An Updated Database of Long Non‐Coding RNA‐Associated Diseases,” Nucleic Acids Research 52 (2024): D1365–D1369. 37819033 10.1093/nar/gkad828 PMC10767967

[25] L. Liu , W. Michowski , A. Kolodziejczyk , and P. Sicinski , “The Cell Cycle in Stem Cell Proliferation, Pluripotency and Differentiation,” Nature Cell Biology 21 (2019): 1060–1067. 31481793 10.1038/s41556-019-0384-4 PMC7065707

[26] W. Yang , R. He , H. Qu , et al., “FXYD3 Enhances IL‐17A Signaling to Promote Psoriasis by Competitively Binding TRAF3 in Keratinocytes,” Cellular & Molecular Immunology 20 (2023): 292–304. 36693922 10.1038/s41423-023-00973-7 PMC9971024

[27] X. Zhou , Y. Chen , L. Cui , Y. Shi , and C. Guo , “Advances in the Pathogenesis of Psoriasis: From Keratinocyte Perspective,” Cell Death & Disease 13 (2022): 81. 35075118 10.1038/s41419-022-04523-3 PMC8786887

[28] F. Kopp and J. T. Mendell , “Functional Classification and Experimental Dissection of Long Noncoding RNAs,” Cell 172 (2018): 393–407. 29373828 10.1016/j.cell.2018.01.011 PMC5978744

[29] R. Gupta , R. Ahn , K. Lai , et al., “Landscape of Long Noncoding RNAs in Psoriatic and Healthy Skin,” Journal of Investigative Dermatology 136 (2016): 603–609. 27015450 10.1016/j.jid.2015.12.009 PMC5546103

[30] S.‐W. Gao and F. Liu , “Novel Insights Into Cell Cycle Regulation of Cell Fate Determination,” Journal of Zhejiang University. Science. B 20 (2019): 467–475. 31090272 10.1631/jzus.B1900197 PMC6568219

[31] H. X. Chao , C. E. Poovey , A. A. Privette , et al., “Orchestration of DNA Damage Checkpoint Dynamics Across the Human Cell Cycle,” Cell Systems 5 (2017): 445–459.e5. 29102360 10.1016/j.cels.2017.09.015 PMC5700845

[32] L. Pasquali , A. Srivastava , F. Meisgen , et al., “The Keratinocyte Transcriptome in Psoriasis: Pathways Related to Immune Responses, Cell Cycle and Keratinization,” Acta Dermato‐Venereologica 99 (2019): 196–205. 30320872 10.2340/00015555-3066

[33] R. Ahn , R. Gupta , K. Lai , N. Chopra , S. T. Arron , and W. Liao , “Network Analysis of Psoriasis Reveals Biological Pathways and Roles for Coding and Long Non‐Coding RNAs,” BMC Genomics 17 (2016): 841. 27793094 10.1186/s12864-016-3188-y PMC5084355

[34] J. Lin , X. Li , F. Zhang , L. Zhu , and Y. Chen , “Transcriptome Wide Analysis of Long Non‐Coding RNA‐Associated ceRNA Regulatory Circuits in Psoriasis,” Journal of Cellular and Molecular Medicine 25 (2021): 6925–6935. 34080300 10.1111/jcmm.16703 PMC8278092

[35] Z. Cheng , J. Guo , L. Chen , N. Luo , W. Yang , and X. Qu , “Knockdown of EHF Inhibited the Proliferation, Invasion and Tumorigenesis of Ovarian Cancer Cells,” Molecular Carcinogenesis 55 (2016): 1048–1059. 26258986 10.1002/mc.22349

[36] X. Zhang , J. Yao , H. Shi , B. Gao , and L. Zhang , “LncRNA TINCR/microRNA‐107/CD36 Regulates Cell Proliferation and Apoptosis in Colorectal Cancer via PPAR Signaling Pathway Based on Bioinformatics Analysis,” Biological Chemistry 400 (2019): 663–675. 30521471 10.1515/hsz-2018-0236

[37] L. Yang , X. Fan , T. Cui , E. Dang , and G. Wang , “Nrf2 Promotes Keratinocyte Proliferation in Psoriasis Through Up‐Regulation of Keratin 6, Keratin 16, and Keratin 17,” Journal of Investigative Dermatology 137 (2017): 2168–2176. 28576737 10.1016/j.jid.2017.05.015

